# How and when is academic stress associated with mobile phone addiction? The roles of psychological distress, peer alienation and rumination

**DOI:** 10.1371/journal.pone.0293094

**Published:** 2024-02-12

**Authors:** Yanhong Zhang, Miao Han, Shuailei Lian, Xiaoxuan Cao, Lei Yan

**Affiliations:** 1 Department of Psychology, College of Education and Sports Sciences, Yangtze University, Jingzhou, China; 2 Social Psychology Research Center of Yangtze University, Jingzhou, China; The World Islamic Sciences and Education University, JORDAN

## Abstract

**Background:**

Mobile phone addiction has a high detection rate among adolescents and is thought to be related to academic stress. However, the underlying mechanisms in this relation were still unclear. The present study tested the mediating role of psychological distress and the moderating roles of peer alienation and rumination in the relationship between academic stress and mobile phone addiction.

**Methods:**

A total of 742 middle school students were recruited to complete measures of academic stress, psychological distress, mobile phone addiction, peer alienation, rumination, and demographic variables. Regression-based statistical mediation and moderation were conducted using the PROCESS macro for SPSS.

**Results:**

The results indicated that academic stress was significantly and positively associated with mobile phone addiction, and this link could be mediated by psychological distress. Moreover, this indirect effect was moderated by both peer alienation and rumination. Specifically, the mediating effect of psychological distress was stronger for adolescents with higher levels of peer alienation or adolescents with higher levels of rumination, as well as those with both higher levels of peer alienation and rumination.

**Conclusion:**

The findings of this study enrich our understanding of how and for whom academic stress is correlated with mobile phone addiction. Education experts and parents should pay special attention to adolescents suffering from academic stress, especially those with higher peer alienation and rumination, and help them get rid of mobile phone addiction.

## Introduction

With the development of information technology, mobile phone as one of the most popular mobile Internet terminals, has a high penetration rate over the world. According to the survey of China Internet Information Center, China had 1.047 billion mobile phone users as of June 2022, with the group of adolescents aged 10–19 years comprising 13.5% and a number as high as 141 million [1]. The portability of mobile phones has not only enhanced the user stickiness of the mobile phone but also shaped the habit of using mobile phones anytime and anywhere and even led to mobile phone addiction. Mobile phone addiction, also known as mobile phone dependence or problematic mobile phone use, refers to excessive dependence on mobile phones in daily life, resulting in serious damage to psychological and social functions [2–4]. The current situation of adolescents using mobile phones mainly focuses on social fun, leisure activities, and information search [5]. Adolescence is a critical period for behavioral change [6], and low self-control levels and strong curiosity for new things among adolescents make it easy to cause problems when using mobile phones [7, 8]. Previous studies have found that academic stress in adolescents is not only related to their psychological distress such as anxiety and depression [9, 10], but also an important proximal risk predictor of mobile phone addiction behavior [11]. And peer relationships (e.g. peer alienation) [28] and rumination [28] are also closely related to mobile phone addiction. It can be seen that mobile phone addiction has become an important inducement for adolescents to fall into emotional and behavioral problems [8–17]. To shelter adolescents from being addicted to mobile phones, researchers suggested that we should pay close attention to the psychological mechanism and susceptible factors of mobile phone addiction among the adolescent population. Therefore, previous studies have explored the formation mechanism of mobile phone addiction from different perspectives, such as personality traits [18], emotion [19], family environment factors [20], and interpersonal relationship quality [21]. However, one question warrants further exploration: Will the interaction of individual personality, emotions, and interpersonal relationships lead to mobile phone addiction under the influence of environmental factors? To address this question, the interactive effects of stress, peer environment, and individual factors will be employed in this study to investigate the formation mechanism of mobile phone addiction in adolescents. This will contribute to an improved understanding of the development of mobile phone addiction in adolescents and provide scientifically sound and practical recommendations for the prevention and intervention of mobile phone addiction.

### Academic stress and mobile phone addiction

Life stress was also considered to be an important inducement of mobile phone addiction [22, 23]. Academic stress, as one of the most important stressors for Chinese adolescents, refers to the psychological pressure and tension caused by academic tasks, mainly including the psychological pressure caused by academic performance, competition with classmates, and the expectations of parents and teachers [24]. Previous studies have also shown that academic stress was positively correlated with adolescent mobile phone addiction, that was, the higher the perceived level of academic stress, the higher the level of mobile phone addiction [8, 25]. Moreover, general strain theory demonstrated that stress or strain is the key cause of individuals’ behavioral problems [9]. Long-term stressful experiences will consume individuals’ self-control resources and reduce their levels of self-control [9, 22]. Self-control failure induced by low self-control ability has been considered the main reason for individuals’ deviant behaviors, such as mobile phone addiction [26]. Mobile phone-based leisure and entertainment activities have become a popular way for individuals to cope with stressful events or relieve stress. Therefore, we put forward:

**Hypothesis 1**: Academic stress will positively predict mobile phone addiction.

### Psychological distress as a mediator

Psychological distress refers to the presence of a nonspecific negative mental health state consisting of multidimensional constructs [26]. It is mainly composed of symptoms of depression, anxiety, and stress [8]. As an internalizing problem, psychological distress may be an important bridge between academic stress and externalizing problems (e.g. mobile phone addiction). The positive correlation between academic stress and psychological distress is self-evident. According to the general strain theory, stressful experiences, such as academic stress, may cause individuals involved in depression, anxiety, and other emotional problems [9, 27]. Empirical studies demonstrated that there is a close relationship between academic stress and psychological distress. For example, academic stress is positively correlated with depression [27], and positively correlated with anxiety [28, 29], academic stress significantly increases psychological stress [30], and promotes the negative mental health of adolescents, such as anxiety and depression levels [31]. Given that psychological distress is a comprehensive index reflecting the levels of depression, anxiety, and stress [8]. We infer that there may be a significant positive correlation between academic stress and psychological distress.

Psychological distress may also be an important predictor of mobile phone addiction. Based on the inference of Young and Kimberly [32] on the causes of Internet addiction, previous researchers generally believed that adolescents’ impulsive behaviors (e.g., mobile phone addiction) can be regarded as an unhealthy means to cope with psychological distress [33, 34]. The compensatory internet use theory (CIUT) also holds that using (or overusing) technology as a compensatory behavior can not only make up for the lack of psychological needs but also help individuals alleviate the negative emotional experience [35]. Specifically, adolescents suffer from psychological distress such as anxiety, depression, and stress, and when this psychological distress leads to unmet psychological needs such as social, entertainment, and identity, they will switch to the excessive use of mobile phones to obtain compensation for their psychological needs [36]. Numerous studies on mobile phone addiction have revealed that individuals addicted to mobile phones are more likely to present symptoms of psychological distress than those who are not addicted to mobile phones [31, 33, 37]. Psychological distress has also been found to be positively associated with mobile phone addiction [38]. Empirical research indicates that psychological distress plays a mediating role in loneliness preference and mobile phone addiction [39], and psychological distress plays a mediating role in the process of physical exercise affecting mobile phone addiction [40]. Therefore, to alleviate psychological distress, individuals who experience depression, anxiety, and stress may habitually devote themselves to the haven of mobile phones, and thus become addicted to mobile phones.

Above all, we put forward:

**Hypothesis 2:** Psychological distress will mediate the association between academic stress and mobile phone addiction.

### The potential individual differences in the proposed mediation model

Although academic stress is an important risk factor for psychological distress and mobile phone addiction, it may exhibit different degrees of effect on psychological distress and mobile phone addiction for different adolescents. In other words, the adverse effects of academic stress on psychological distress or mobile phone addiction may vary with psychological traits (e.g. rumination) or peer environment factors (e.g. peer alienation). The individual-context interaction theory points out that the internalizing or externalizing problems are the result of the interaction between psychological traits (e.g. rumination) and contextual factors (e.g. peer environment factors) in a dynamic way [41]. Rumination, as a negative psychological trait, has been proven to accelerate the process of other factors that cause internalizing problems [42]. Peer alienation, as a negative environmental factor, has been proven to be associated with internalizing or externalizing problems [43, 44]. However, previous studies only examined the individual differences in the relationship between academic stress, psychological distress, and mobile phone addiction from the perspective of psychological traits (academic resilience) [8]. The role of peer environment factors in the relationship between academic stress, psychological distress, and mobile phone addiction was ignored. The interaction between psychological trait factors and environmental factors in this relationship has not received due attention. Therefore, to comprehensively understand the mediating and conditional factors of academic stress leading to mobile phone addiction, it is necessary to examine the moderating roles of psychological traits (e.g. rumination) and peer environment factors (e.g. peer alienation) simultaneously in the relationship among academic stress, psychological distress and mobile phone addiction in an integrated model. Thus, we supposed that:

**Hypothesis 3:** The relationship among academic stress, psychological distress, and mobile phone addiction may vary with individuals’ levels of peer alienation and rumination.

### Peer alienation as a moderator

The influence of peer environment on adolescents’ psychosocial adaptation is more profound and comprehensive in adolescence. Peer alienation, as an indicator of peer environment, refers to the experience and state of adolescents being isolated from peer groups [45]. Peer alienation will not only affect adolescents’ self-evaluation but also affect the perception of peer-supportive response [46]. It also has been proved to be negatively associated with adaptive behavior, such as prosocial behavior, and be positively correlated to internalizing and externalizing problems, such as depressive symptoms, anxiety, cyber deviant behaviors, and addictive behaviors [43, 44, 47]. According to the developmental contextualize theory [48], different developmental contexts and their interactions determine different internalizing and externalizing consequences. Specifically, adaptive environmental factors and their interactions may optimize the potential outcomes of psychological development, whereas adverse environmental factors and their interactions may exacerbate the potential negative consequences of psychological development. Academic stress and peer alienation are the most common adverse environmental factors of adolescents [44, 49], thus they may interact with each other and induce more internalizing and externalizing problems. The negative effects of academic stress and peer alienation on psychosocial adaptation may show a superposition effect. That is, adolescents with higher levels of peer alienation may be surrounded by more internalizing and externalizing problems when experiencing academic stress than those with lower levels of peer alienation. Previous studies considered that individuals suffering from higher levels of peer alienation often have more sense of anxiety sensitivity and worry, helplessness, and loneliness than those with lower levels of peer alienation [50]. This makes them lose confidence in actively taking measures to get rid of the adverse effects of stressful life events and thus makes them involved in more internalizing and externalizing problems. Besides, high-quality relationship with peers is an important source of satisfying adolescents’ psychological needs (e.g. need for belonging) in adolescence [51]. Adolescents whose psychological needs are satisfied can cope with stressful life events more effectively [52]. Therefore, adolescents with lower levels of peer alienation can cope with academic stress more effectively, and thus have fewer internalizing and externalizing problems. In contrast, adolescents with higher levels of peer alienation generally do not have enough psychological resources to cope with academic stress and are thus involved in more internalizing and externalizing problems. Above all, we supposed that:

**Hypothesis 3a**: Peer alienation may moderate the association between academic stress and psychological distress or mobile phone addiction.

### Rumination as another moderator

Different from peer alienation, rumination was known as a negative personality trait. It could not only directly threaten adolescents’ mental health, but also could act as a catalyzer in the process of potential environmental risk factors leading to internalizing and externalizing problems. Rumination, as a maladaptive response style, refers to the tendency of repeatedly thinking about the negative experience and its causes or potential adverse consequences without thinking about the constructive strategies to improve or solve the negative or stressful life events that they are going through [53]. Prior studies have shown that rumination was associated with a wide range of non-adaptive psychological and behavioral consequences, such as psychological distress, impulsive buying, and problematic smartphone use [8, 34, 54, 55]. Although rumination represents a common response style, different people show different levels of rumination, which may result in different degrees of externalizing and internalizing problems [42, 56]. Rumination, thus, was also regarded as a relatively stable negative psychological trait that could aggravate the relationship between stress and externalizing or internalizing problems [57]. According to response style theory [53, 56], individuals with higher levels of rumination often think about stressful life events and their causes or potential adverse consequences repeatedly without thinking about constructive strategies to improve or solve the stressful life events. This tendency or stress response style enhances the possibility of their emotional and behavioral problems, such as higher levels of psychological distress or mobile phone addiction. On the contrary, individuals with lower levels of rumination tend to put aside the causes or potential adverse consequences of stressful life events. Thus, they are used to thinking about constructive strategies and ways as well as taking action or making efforts to solve stressful life events. Empirical research has also shown that rumination could deteriorate the adverse effects of various non-adaptive factors on individuals’ externalizing or internalizing problems [34, 42, 57]. For instance, Genet and Siemer [42] found that individuals with higher levels of rumination have more negative moods than those with lower levels of rumination when suffering from stressful life events. Liu, He, and Li [34] also demonstrated that rumination could moderate the link between upward social comparison on social network sites and impulse buying, with the effect of upward social comparison on social network sites on impulse buying being stronger for individuals with higher levels of rumination.

As far as this study was concerned, individuals with higher levels of rumination are more likely to be involved in compulsive thinking about the causes or potential adverse consequences of academic stressful events and thus get involved in more psychological distress unconsciously. Besides, given that mobile phone-based leisure and entertainment activities have become a popular way for individuals to avoid stressful life events or relieve negative emotional consequences, individuals with higher levels of rumination may also be addicted to mobile phones to cope with academic stress. Therefore, it is reasonable for us to put forward:

**Hypothesis 3b:** The association between academic stress and psychological distress or mobile phone addiction could be moderated by rumination.

### The combined effect of peer alienation and rumination

According to the developmental contextualize theory [48], internalizing or externalizing problems are the results of many environmental factors (e.g. academic stress, and peer alienation) and their interactions. However, environmental factors (e.g. academic stress, and peer alienation) and their interactions could only explain the general character, whereas ignoring the individual differences of the emergence or development of internalizing or externalizing problems. Therefore, the individual-context interaction theory pointed out that the effects of environmental factors (e.g. academic stress, peer alienation) and their interactions on internalizing or externalizing problems may vary with different levels of psychological traits (e.g. rumination) [41]. It will be more conducive for us to reveal the emergence or development of internalizing or externalizing problems from an integrated perspective of environmental factors and psychological traits, compared with a single perspective of the environmental factors or psychological traits. Thus, what is the combined effect of peer alienation and rumination in the relationship between academic stress, psychological distress, and mobile phone addiction? To address this question, based on testing the moderating effects of peer alienation and rumination, respectively, the present study will also examine the combined moderating effects of peer alienation and rumination in the link among academic stress, psychological distress, and mobile phone addiction.

Thus, we put forward:

**Hypothesis 3c:** The adverse effect of academic stress on psychological distress or mobile phone addiction may be stronger for individuals with higher levels of peer alienation and rumination than those with lower levels of peer alienation and rumination.

## The present study

Reviewing the previous literature, we found that in the previous studies, there was less noticed in the same study to investigate the interaction of stress, peer environment, and individual factors on mobile phone addiction. However, previous reviews have shown that mobile phone addiction was the result of multiple factors. Therefore, to fill this gap, this study attempts to explore the impact of academic stress on adolescent mobile phone addiction and investigate the potential mechanisms of psychological distress, peer alienation, and rumination between academic stress and mobile phone addiction. This study will not only help to improve our understanding of the underlying mechanisms and boundary conditions in the process of academic stress linked to mobile phone addiction but also provide useful suggestions for educators and parents to guide adolescents to treat or release academic stress more appropriately, establish good peer relationship and reduce rumination response, to promote their mental health and behavioral adaptation.

**Fig 1:**
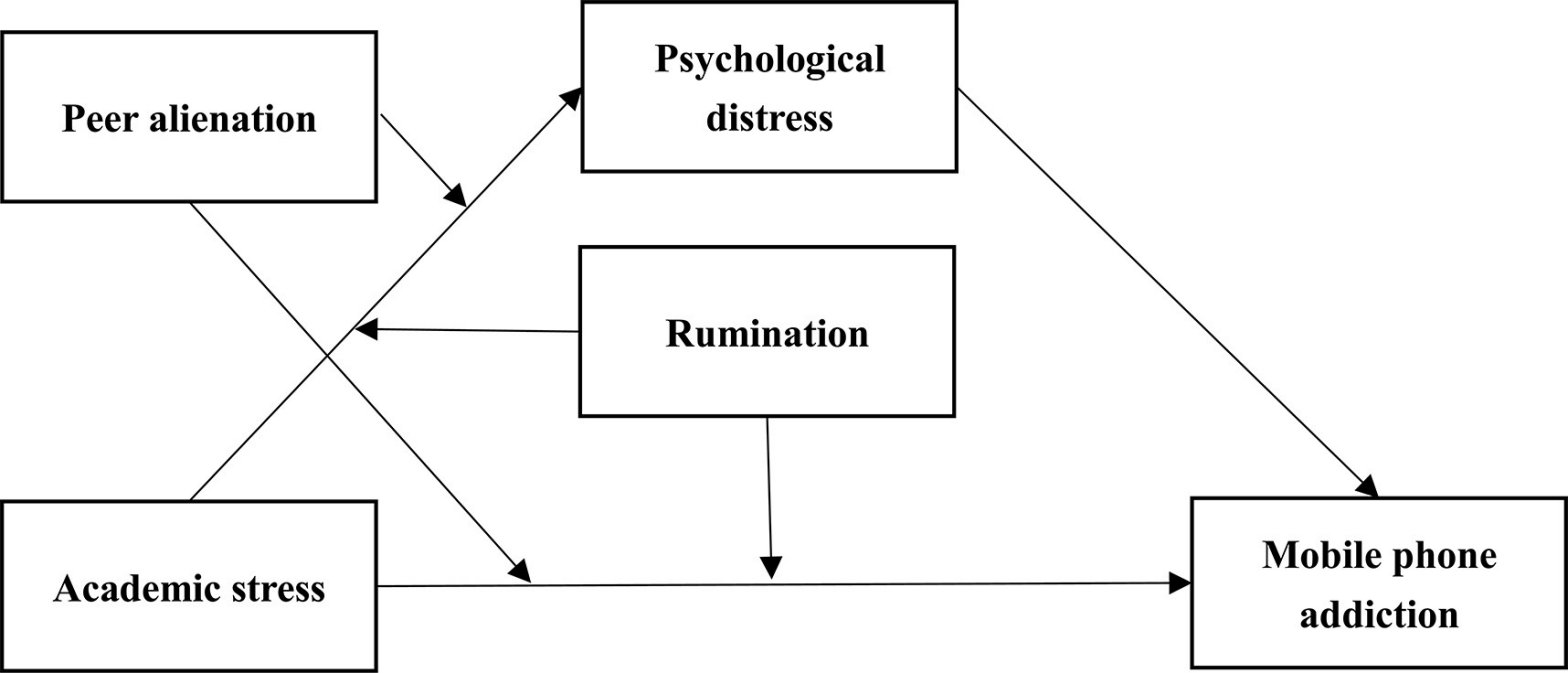
The proposed moderated mediation model.

Based on existing conclusions and research hypothesis, a moderated mediation model was constructed in the current study. The complete hypothesized model is presented in Fig 1. The model can help us shed light on the mediation and moderation mechanisms underlying the relationship between academic stress and mobile phone addiction.

## Methods

### Participants and procedure

In this study, we used G*Power3.1.9.7 to calculate the sample size [58], the calculated parameters including Tails = two, Effect size = 0.2, α err prob = 0.01, Power (1-β) = 0.99, the calculated sample size is 581. Considering the invalid response rate of the subjects, assuming that the invalid response rate is 20%, 581 / (1–0.2) = 726 questionnaires should be sent out. Data collection was conducted from May 24–28, 2021. The inclusion criteria of this study were full-time middle school students, who fill in within 10–30 minutes, and voluntarily participate in the survey. The exclusion criteria were that the filling time was less than 10 minutes, the answers were incomplete, and the answers were given regularly. A total of 760 middle school students from two junior middle schools were recruited to participate in the survey through convenience sampling, and 742 valid questionnaires were collected, with an effective rate of 97.63%. Both the two junior middle schools are typical junior middle schools from two cities located in central China. The average age of all participants was 12.90 (*SD* = 0.892), with ages ranging from 11 to 15. 54.6% of them were girls. All of the participants were from three grades, with 342 Grade 7 students (46.1%), 272 Grade 8 students (36.7%), and 128 Grade 9 students (17.2%). It should be noted that according to the principle of voluntary participation, some Grade 9 students refused the invitation to the survey due to the pressure of entrance examination from junior middle school to senior middle school. The Research Ethics Committee of the Psychology Department at Yangtze University gave its ethical approval for this study, and all research procedures adhered to ethical guidelines. After obtaining verbal informed consent, investigators encouraged the participants to respond truthfully and emphasized the principles of anonymity, independence, and confidentiality of this survey. All of the participants completed the paper-and-pencil survey measuring their academic stress, psychological distress, mobile phone addiction, peer alienation, rumination, and demographic variables within 30 minutes in their classroom.

### Measurements

#### Academic stress

The academic stress subscale of the Adolescent Self-rating Life Events Checklist (ASLEC) was adopted to assess participants’ academic stress [59]. The original ASLEC was used to assess the frequency and intensity of daily stress experienced by participants. It consists of 27 items, including interpersonal relationships, academic stress, punishments, loss of relatives and friends and property, health and adaptation problems, and others [60]. The academic stress subscale contains 5 items (e.g., “a heavy load of study”) and requires participants to evaluate whether or not they had experienced the academic stressful life events described in each item. If they answered “yes” on these items, they were further requested to assess the influence of academic stressful life events on a 5 Likert-type scale. The index of confirmatory factor analysis (CFA) suggested a good fit: *χ*^*2*^*/df* = 2.59, RMSEA = 0.05, AGFI = 0.98, NFI = 0.98, GFI = 0.99, IFI = 0.99, TLI = 0.97, CFI = 0.99. In this study, Cronbach’s α of the ASLEC was 0.909, and Cronbach’s α for academic stress subscale was 0.635, which was acceptable according to DeVellis [61].

#### Psychological distress

The participants’ psychological distress was measured by the depressive symptom subscales and anxiety symptoms subscales, which were derived from the Chinese version of the Depression Anxiety Stress Scale-21 (DASS-21) [62]. It contains 3 dimensions of depression, anxiety, and stress, and each dimension has 7 items (e.g., “I found it is hard to wind down”). These 14 items require participants to respond on a scale from 0 (did not apply to me at all) to 3 (applied to me very much or most of the time). In this study, the index of CFA suggested a good fit: *χ*^*2*^*/df* = 2.82, RMSEA = 0.05, AGFI = 0.92, NFI = 0.91, GFI = 0.94, IFI = 0.94, TLI = 0.93, CFI = 0.94. Cronbach’s α for this scale was 0.877.

#### Peer alienation

The peer alienation subscale of the Inventory of Parent and Peer Attachment (IPPA) was employed to assess participants’ experience of peer alienation [63]. It contains three dimensions of trust, communication, and alienation, among which there are 10, 10, and 8 items in the parent attachment questionnaire and 10, 8, and 7 items respectively in the peer attachment questionnaire. The peer alienation subscale consists of seven items (e.g., “My friends seem to annoy me for no reason”). All of the participants were required to rate these seven items on a five-point Likert-type scale ranging from 1 (never) to 5 (always). All of the responses were averaged after reversing the reverse-scored items to form a score, with higher scores meaning higher levels of peer alienation. The index of CFA suggested a good fit: *χ*^*2*^*/df* = 3.32, RMSEA = 0.06, AGFI = 0.96, NFI = 0.93, GFI = 0.98, IFI = 0.95, TLI = 0.92, CFI = 0.95. In this study, Cronbach’s α of the IPPA was 0.909, and Cronbach’s α for the peer alienation subscale in the present study was 0.626, which was acceptable according to DeVellis [61].

#### Rumination

The Chinese short version of the Ruminative Response Scale [64], revised from Treynor, Gonzalez, and Nolen-Hoeksema [53], was used to measure the ruminative response of participants. It has two factors reflective thinking and compulsive thinking, each with 5 items. This scale consists of 10 items (e.g., “Think about how sad you feel”). All of the items require participants to assess their ruminative response on a Likert-type scale ranging from 1 (never) to 4 (always). Higher scores on this scale indicate a higher tendency to ruminative response tendency in daily life. The index of CFA suggested a good fit: *χ*^*2*^*/df* = 2.91, RMSEA = 0.05, AGFI = 0.96, NFI = 0.95, GFI = 0.98, IFI = 0.96, TLI = 0.95, CFI = 0.96. In the current study, Cronbach’s α was 0.798.

#### Mobile phone addiction

The Mobile Phone Addiction Index was used to assess the participants’ mobile phone addiction (MPAI) [65]. It has four dimensions: uncontrollability, withdrawal, escapism, and inefficiency. Each dimension has 7, 5, 3, and 2 items respectively. This scale includes seventeen items (e.g., “You have attempted to spend less time on your mobile phone but are unable to”). All these items require participants to respond on a Likert-type scale ranging from 1 (never) to 5 (always) to assess their mobile phone addiction tendencies. All of the responses on each item were averaged to form a measure of students’ mobile phone addiction, with higher scores indicating greater mobile phone addiction tendency. The index of CFA suggested a good fit: *χ*^*2*^*/df* = 2.72, RMSEA = 0.06, AGFI = 0.91, NFI = 0.93, GFI = 0.94, IFI = 0.95, TLI = 0.93, CFI = 0.95. Cronbach’s α was 0.890.

#### Control variables

Given that gender, age, grade, and years of mobile phone use were closely related to the main variables of this study in previous studies, they were included as control variables in the present study [8, 66].

### Statistical analyses

Before testing the proposed moderated mediation model, descriptive statistics were adopted to examine the means and standard deviations for academic stress, mobile phone addiction, psychological distress, peer alienation, and rumination. The normality test indicated that the skewness values and kurtosis values of the data for each variable were within the allowed range of normal distribution, and there were no extreme nonnormality problems, which allowed relevant statistical analysis of the data. The bivariate associations for all of the research variables were examined by Pearson correlation analysis. The gender differences for all research variables were tested by Independent-sample t-test. The Grade-Level differences for all research variables were determined by One-way ANOVA. On this basis, the SPSS macro PROCESS (model 10) by Hayes [67] was adopted to investigate the mediating role of psychological distress and the moderating roles of peer alienation and rumination in the relationship between academic stress and mobile phone addiction. Model 10 of the SPSS macro PROCESS supposed that the direct effect of the independent variable on the dependent variable could be mediated by one mediator. It also supposed that both the direct effect of the independent variable on the dependent variable and the effect of the independent variable on the mediator could be moderated by two parallel moderators. The SPSS macro PROCESS suggested by Hayes [67] has been employed to test the moderated mediation model by many studies and showed high statistical testability [8, 17, 23]. Moreover, simple slope analysis, suggested by Aiken and West [68], was used to dissect the significant interaction effects.

## Results

### Common method biases test

This study used the exploratory factor analysis recommended by Zhou and Long [69] to test the possible common method biases. Using SPSS26.0, all items of each questionnaire were taken as all items of exploratory factor analysis, and 22 factors with eigenvalues greater than 1 were analyzed. The interpretation rate of the first common factor was 16.05%, far less than 40%, indicating that there were no serious common method biases in the data in this study.

### Preliminary analyses

Descriptive statistics and Pearson correlations (Table 1) were conducted in SPSS 26.0, including means, standard deviations, and Pearson correlations. The results showed that the variables in this study were closely related, which means they were suitable for further analysis.

**Table 1:** Descriptive statistics and interrelations among all of the observed variables.

*Variables*	*M*	*SD*	1	2	3	4	5
1. Academic stress	1.694	0.841	1				
2. Psychological distress	0.801	0.530	0.466**	1			
3. Peer alienation	2.571	0.630	0.257**	0.398**	1		
4. Rumination	2.240	0.560	0.165**	0.255**	0.175**	1	
5. Mobile phone addiction	2.320	0.773	0.370**	0.468**	0.280**	0.178**	1

Note. *N* = 742.

**p< 0.01

*p< 0.05.

Table 2 shows the gender differences and grade-level differences in the observed variables. The results of the Independent-sample t-test indicated that all of the research variables showed no gender differences (See Table 2). The results of One-way ANOVA indicated that all of the research variables showed no Grade-Level differences (See Table 2). Because of prior evidence suggesting gender, age, grade, and years of mobile phone use were related to mobile phone addiction, we included gender, age, grade, and years of mobile phone use as control variables in testing for all mediating and moderating models.

**Table 2:** The gender differences and Grade-Level differences of the observed variables.

*Variables*	*M*	Academic stress	Psychological distress	Peer alienation	Rumination	Mobile phone addiction
Gender	Male	1.682±0.876	0.801±0.517	2.555±0.642	2.236±0.547	2.351±0.769
	Female	1.705±0.806	0.801±0.543	2.588±0.619	2.244±0.574	2.299±0.778
	*t*	-0.382	-0.003	-0.716	-0.179	1.070
Grade	Grade 7	1.721±0.830	0.845±0.536	2.571±0.657	2.197±0.542	2.300±0.745
	Grade 8	1.618±0.851	0.772±0.543	2.598±0.634	2.290±0.586	2.330±0.799
	Grade 9	1.783±0.843	1.801±0.530	2.513±0.542	2.248±0.560	2.355±0.797
	*F*	2.008	2.213	0.778	2.125	0.272

Note. *N* = 742.

**p< 0.01

*p< 0.05.

### Testing for the proposed moderated mediation model

The SPSS macro program PROCESS (model 10) was performed to test the proposed moderated mediation model. The results are shown in Table 3.

**Table 3:** Regression results for the conditional indirect effects (moderated mediation).

*Model*										
*Model 1*: *Total effect model*										
*R*	*R* ^*2*^	*F*		*df*_*1*_	*df*_*2*_	*p*	*B*	*SE*	*t*	*p*
0.410	0.168	29.725		5	741	< 0.001				
Constant							0.930	0.582	1.598	>0.05
Gender							-0.047	0.052	-0.893	>0.05
Age							0.062	0.050	1.250	>0.05
Grade							-0.042	0.060	-0.695	>0.05
Years of mobile phone use							0.071***	0.014	4.887	< 0.001
Academic stress							0.321***	0.031	10.290	< 0.001
*Model 2*: *Mediator variable model*										
*R*	*R* ^*2*^	*F*		*df*_*1*_	*df*_*2*_	*p*	*B*	*SE*	*t*	*p*
0.591	0.349	36.464		9	732	< 0.001				
Constant							0.978	0.379	2.580	<0.05
Gender							-0.012	0.032	-0.392	>0.05
Age							-0.010	0.033	-0.320	>0.05
Grade							-0.047	0.038	-1.227	>0.05
Years of mobile phone use							0.015	0.009	1.755	>0.05
Academic stress							0.201	0.021	9.457	<0.001
Peer alienation							0.236	0.038	6.168	<0.001
Academic stress × Peer alienation							0.094	0.044	2.118	<0.05
Rumination							0.136	0.032	4.193	<0.001
Academic stress × Rumination							0.082	0.041	2.013	<0.05
*Model 3*: *Dependent variable model*										
*R*	*R* ^*2*^	*F*		*df*_*1*_	*df*_*2*_	*p*	*B*	*SE*	*t*	*p*
0.533	0.284	27.278		10	731	< 0.001				
Constant							1.279*	0.545	2.345	<0.05
Gender							-0.053	0.049	-1.067	>0.05
Age							0.044	0.047	0.936	>0.05
Grade							0.005	0.056	0.084	>0.05
Years of mobile phone use							0.060***	0.014	4.191	<0.001
Psychological distress							0.472***	0.066	7.133	< 0.001
Academic stress							0.149***	0.036	4.141	< 0.001
Peer alienation							0.125**	0.045	2.798	< 0.01
Academic stress × Peer alienation							0.035	0.047	0.744	>0.05
Rumination							0.036	0.045	0.796	>0.05
Academic stress × Rumination							0.010	0.054	0.189	>0.05
*Conditional indirect effect analysis at values of peer alienation and rumination (M ± SD)*										
Peer alienation			Rumination				*B*	*SE*	LLCI	ULCI
*M–* 1*SD (1*.*941)*			*M–* 1*SD* (1.680)				0.045	0.017	0.015	0.084
*M*– 1*SD* (1.941)			*M* (2.240)				0.067	0.017	0.038	0.106
*M*– 1*SD* (1.941)			*M* + 1*SD* (2.800)				0.089	0.023	0.049	0.141
*M* (2.571)			*M*– 1*SD* (1.680)				0.073	0.018	0.044	0.113
*M* (2.571)			*M* (2.240)				0.095	0.016	0.068	0.129
*M* (2.571)			*M* + 1*SD* (2.800)				0.117	0.020	0.081	0.163
*M* + 1*SD* (3.201)			*M*– 1*SD* (1.680)				0.101	0.025	0.058	0.156
*M* + 1*SD* (3.201)			*M* (2.240)				0.123	0.022	0.084	0.171
*M* + 1*SD* (3.201)			*M* + 1*SD* (2.800)				0.144	0.024	0.102	0.197

Note. *N* = 742. Unstandardized regression coefficients are reported. Bootstrap sample size = 5000. LL = low limit, CI = confidence interval, UL = upper limit.

*p< 0.05.

**p< 0.01.

***p< 0.001.

As expected, academic stress positively predicted psychological distress (*B* 0.201, *p* < 0.001) and mobile phone addiction (*B* = 0.149, *p* < 0.001). Psychological distress positively predicted mobile phone addiction (*B* = 0.472, *p* < 0.001). Furthermore, the significance of the indirect effect of academic stress on mobile phone addiction via psychological distress was tested by the Sobel test. The results indicated that psychological distress significantly mediated the relationship between academic stress and mobile phone addiction (*z* = 5.729, *p* < 0.001), indirect effect = 0.156, SE = 0.020, 95%CI = [0.117, 0.197]. These results provided persuasive evidence that academic stress was associated with an increase in mobile phone addiction and this link was mediated by psychological distress. Thus, Hypothesis 1 and 2 were supported.

To examine Hypothesis 2, four interaction effects were examined with PROCESS macro (Model 10) by Hayes [67]. Both the interaction effect of academic stress × peer alienation (*B* = 0.094, *p <* 0.05) and the interaction effect of academic stress × rumination (*B* = 0.082, *p <* 0.05) on psychological distress were all significant in the mediator variable model. Whereas, the interaction effect of academic stress × peer alienation (*B* = 0.035, *p >* 0.05) and the interaction effect of academic stress × rumination (*B* = 0.010, *p >* 0.05) on mobile phone addiction was not significant in the dependent variable model. These findings indicated that the association between academic stress and psychological distress could be moderated by both peer alienation and rumination.

**Fig 2:**
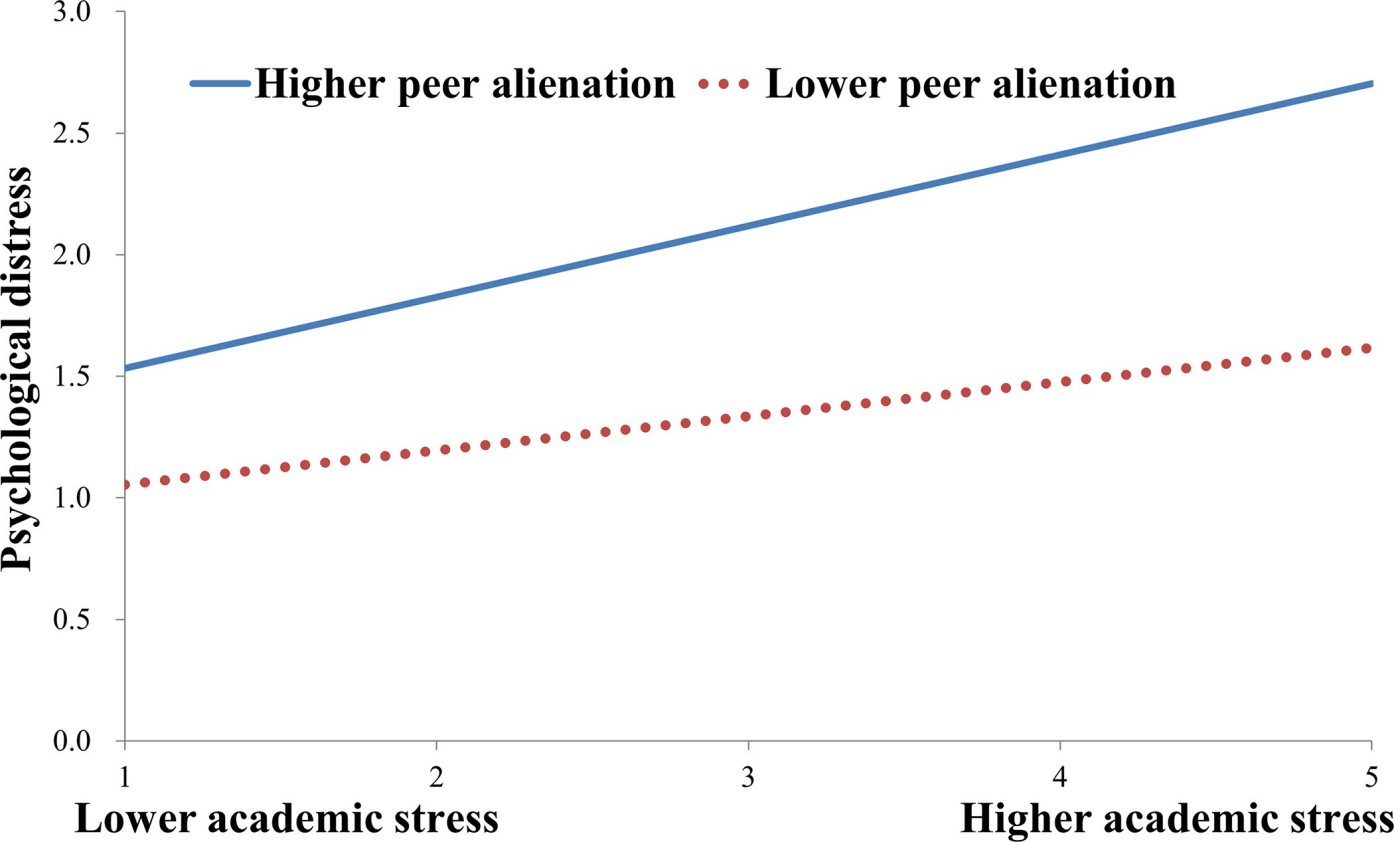
Peer alienation moderates the relationship between academic stress and psychological distress.

**Fig 3:**
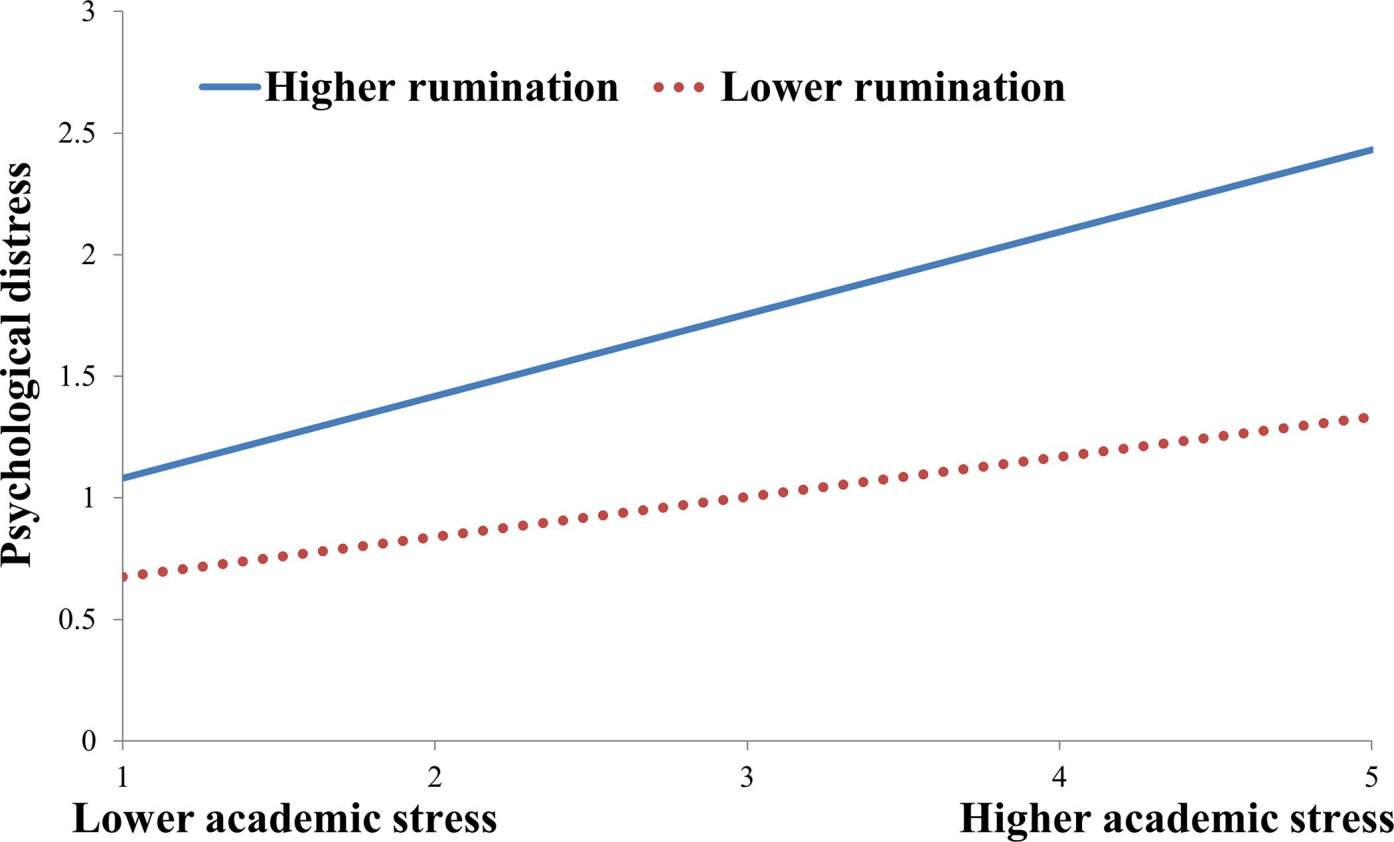
Rumination moderates the relationship between academic stress and psychological distress.

To decompose these two significant interaction effects and explore whether slopes for the high-peer alienation group (1 *SD* above the mean) or high-rumination group (1 *SD* above the mean) were different from slopes for the low-peer alienation group (1 *SD* below the mean) or low-rumination group (1 *SD* below the mean) in the mediator variable model. The results are plotted in Figs 2 and 3. Fig 2 showed that the effect of academic stress on psychological distress was stronger for students suffering from higher levels of peer alienation (*B* = 0.292, *t* = 8.754, *p* < 0.001) than for those suffering from lower levels of peer alienation (*B* = 0.141, *t* = 4,217, *p* < 0.001). As shown in Fig 3, the effect of academic stress on psychological distress was stronger for students with higher levels of rumination (*B* = 0.337, *t* = 12.532, *p* < 0.001) than for those with lower levels of rumination (*B* = 0.164, *t* = 4.686, *p* < 0.001).

Results drawn from the conditional analyses at values of both peer alienation and rumination also indicated that the indirect effects of academic stress on mobile phone addiction via psychological distress were positively and significantly different from zero for all adolescents, no matter what levels of peer alienation and rumination they were at. Moreover, with the increasing values of peer alienation and rumination, the indirect effects of academic stress on mobile phone addiction via psychological distress were increasing. Generally, this indirect effect was stronger for adolescents with higher levels of peer alienation and higher levels of rumination. In other words, adolescents with higher levels of rumination and higher levels of peer alienation reported higher levels of psychological distress and mobile phone addiction when they suffering from academic stress. On the contrary, adolescents with lower levels of rumination and lower levels of peer alienation reported lower levels of psychological distress and mobile phone addiction when suffering from academic stress.

## Discussion

With the popularity of mobile phones among adolescents, adolescents as digital natives have become the main force of mobile phone subscribers and more and more of them are addicted to mobile phones [8, 17, 23]. However, few studies have paid attention to the underlying mechanisms and potential individual differences in the link between academic stress and mobile phone addiction. To fill this gap, this study shed light on the mediating role of psychological distress and the moderating roles of peer alienation and rumination in the relationship between academic stress and mobile phone addiction. The results will be helpful to reveal how academic stress results in mobile phone addiction, and when or for whom the link is stronger. This study enriched our understanding of the underlying mechanisms and boundary conditions of academic stress linked to mobile phone addiction. This study also enlightens us that environmental factors and psychological traits should be considered simultaneously when exploring the relationship between academic stress and internalizing and externalizing problems, which also provides empirical evidence for the prevention of mobile phone addiction in adolescents and interventions against this problem.

### The relation between academic stress and mobile phone addiction

Our findings resonate well with prior results that examined the link between stress and mobile phone addiction [8, 23]. Prior studies demonstrated that life stress is a hotbed of mobile phone addiction [23], and showed that academic stress was the most frequent factor contributing to mobile phone addiction among Chinese adolescents [8, 25]. The results of the present study showed that academic stress was positively associated with mobile phone addiction, which also validated the general strain theory, which holds that stress or strain is the key cause of individuals’ behavioral problems [9, 21]. However, adolescents as individuals on their way to maturity, lack self-control ability when releasing stress or coping with psychological problems using mobile phones [23]. Therefore, long-term academic stress for adolescents would consume individuals’ self-control resources, leading to a lower level of self-control, and would use mobile phones as a way to escape academic stress and thus become addicted to mobile phones.

### The mediating role of psychological distress

The present study further explored the underlying mechanisms in the link between academic stress and mobile phone addiction and revealed the mediating role of psychological distress in this link. The results showed that academic stress was positively associated with mobile phone addiction, and this relation could be mediated by psychological distress (Hypothesis 2 was supported). This result is consistent with previous studies, indicating that the psychological distress caused by academic stress enhances the inevitable relationship between academic stress and mobile phone addiction [8]. This result was also consistent with the compensatory internet use theory (CIUT) [35], which holds that mobile phone addiction has become an inevitable consequence of adolescents relying on mobile phones to cope with stress and relieve psychological distress. The perceived disadvantage of adolescents suffering from academic stress in peer competition can induce depression, anxiety, stress, and unsatisfied psychological needs. Mobile phone use, as a compensatory behavior, has been demonstrated to not only make up for unsatisfied psychological needs but also relax the psychological distress brought by stressors [8, 23]. Therefore, adolescents suffering from academic stress may be addicted to mobile phones due to their habitual behavior of using a mobile phone to alleviate psychological distress. Overall, psychological distress was an underlying mechanism for us to understand how academic stress leads to mobile phone addiction.

### The moderating role of peer alienation

The results of the present study showed that the mediating effect of psychological distress in the link between academic stress and mobile phone addiction was stronger for adolescents with higher levels of peer alienation (Hypothesis 3a was supported partially). This result means that peer alienation, as an adverse peer environmental factor, could aggravate the adverse effect of other factors on internalizing problems. Previous studies have shown that individuals with higher levels of peer alienation tend to have more experience of being isolated, excluded, and abandoned [45]. This makes them enter a state of "adding insult to injury" after experiencing academic stress. The feeling of being isolated, excluded, and abandoned brought by peer alienation and the frustration brought by academic stress have a superimposed adverse impact on their mental health. Besides, positive self-evaluation, high self-esteem, and high self-efficacy are important resources for individuals to cope with stress [70]. However, individuals with higher levels of peer alienation are more likely to have negative self-evaluation, low self-esteem, and low self-efficacy [46]. This makes them lack the necessary resources to deal with the stress smoothly. Moreover, social support from peers is an important resource for adolescents to cope with stress [71, 72]. Regrettably, individuals with higher levels of peer alienation were often unable to get the necessary social support from peer relationships [50, 73]. This makes them lose the protective effect of peer social support and are directly exposed to academic stress. In summary, adolescents with higher levels of peer alienation will have more psychological distress and be more likely to be addicted to mobile phones when suffering from academic stress than those with lower levels of peer alienation.

### The moderating role of rumination

This study demonstrated that the indirect effect of psychological distress in the link between academic stress and mobile phone addiction varied with adolescents’ levels of rumination, with the effect being stronger for adolescents with higher levels of rumination (Hypothesis 3b was supported partially). This result indicated that rumination, as a negative psychological trait, could also act as a catalyzer exacerbating the unfavorable effect of academic stress on adolescents’ mental health. This finding is consistent with the response style theory [56, 74]. The response style theory holds that adolescents with higher levels of rumination may waste most of their attention and time on ruminative thinking about the stress scenarios and the causes of the stress scenarios or the potential adverse consequences continuously and repeatedly, instead of thinking about constructive solutions to stress scenarios and putting them into action in due course of time. They will, of course, pay for their response style and be involved in more psychological distress. This has also been confirmed by empirical research [42]. Moreover, prior studies demonstrated that individuals with higher levels of rumination often have poor attention control ability and can’t switch their attention away from stress scenarios to their advantage, and thus fail to effectively solve stressful events [75, 76]. Therefore, adolescents with higher levels of rumination may have more psychological distress while suffering from academic stress. To sum up, adolescents with higher levels of rumination perceived more psychological distress when suffering from academic stress and thus were more likely to be addicted to mobile phones.

### The combined moderating effects of peer alienation and rumination

A more valuable finding revealed in this study was the combined moderating effects of peer alienation and rumination in the underlying mechanism of academic stress leading to mobile phone addiction, with the indirect effect of psychological distress being stronger for adolescents with both higher levels of peer alienation and rumination (Hypothesis 3c was supported partially). This result indicated that internalizing problems, such as psychological distress, were the consequence of the joint effects of negative environmental factors and negative psychological traits. This result can be explained by individual-context interaction theory, which argues that environmental factors and psychological traits and their interactions jointly determine the consequences of psychological development [41]. According to this theory, positive environmental factors combined with positive psychological traits will present a coupling effect, which could be named as icing on the cake. Positive environmental factors combined with negative psychological traits or negative environmental factors combined with positive psychological traits will show a neutralizing effect, which could be named as fuel in snowy weather. The joint model mentioned above will have a positive effect on psychological adaptation, at least not an adverse effect. Unfortunately, negative environmental factors combined with negative psychological traits will deliver a serious adverse effect on psychological development, which is like snow plus frost. Adolescents with both higher levels of peer alienation and higher levels of rumination may have a higher risk of psychological distress when suffering from academic stress. This was probably because adolescents with both higher levels of peer alienation and higher levels of rumination were more likely to fail in coping with academic stress without necessary peer social support and effective thinking style, and thus were surrounded by more psychological distress.

In addition, it should be treated cautiously that the three-way interaction (Academic stress × Peer alienation × Rumination) was not significant. The reasons why the three interactions are not significant: first, from the perspective of the mathematical statistical principle of regression analysis, the higher the interaction order, the less significant the prediction of the interaction term of the dependent variable; Secondly, from the perspective of data morphology, the more product terms are, the farther the form of the data represented by the product term deviates from the normal, which will lead to its insignificant prediction effect on the dependent variable. Third, from the perspective of multi-collinearity, because the same equation includes academic stress, peer alienation, rumination, the interaction between academic stress and rumination, the interaction between academic stress and peer alienation, and three interaction items of academic stress, peer alienation, and rumination, this artificially increases the possibility of multicollinearity in the regression equation to a certain extent, This may also be the main reasons why the three interactive items do not predict the dependent variable significantly. Researchers or readers should take this non-significant three-way interaction into account when making inferences based on this study.

## Conclusions

In this study, the effects of academic stress on mobile phone addiction in adolescents and the underlying mechanisms are explored. Among them, based on the general strain theory and the compensatory internet use theory, we explored the mediating role of psychological distress in the relationship between academic stress and mobile phone addiction. Based on the developmental contextualize theory and individual-context interaction theory, we explored the moderating role of peer alienation and rumination between academic stress, psychological distress, and mobile phone addiction. This study provided a valuable comprehensive perspective to reveal how and when or for whom academic stress influences adolescents’ mobile phone addiction. However, some limitations should be taken into account when interpreting the results.

First, given that a rigorous causal relationship cannot be drawn from a cross-sectional design, the results of this study cannot be inferred in the framework of causality. A large-scale and long-term longitudinal design should be adopted in future studies to examine the causal relationship between academic stress and mobile phone addiction. A rigorous and ingenious experimental intervention design should be conducted to illustrate the trend of this causal relationship changing with the levels of peer alienation and rumination as well as the combined levels of both peer alienation and rumination. Second, due to the limitation of convenient sampling of statistical samples recruited only in two junior middle schools in central China and the limitation of research funds, we were not allowed to interpret the conclusions of this study in populations with different demographic characteristics or different cultural environments. Moreover, since the convenient self-report questionnaire was employed to collect all the data for this study, the potential social desirability bias will also restrict our inferences about the results of this study. Therefore, future studies should draw multidimensional scaling into research design to enhance the objectivity and preciseness of the original data.

The findings of this study still have important theoretical implications for us to understand the formation mechanisms of mobile phone addiction among Chinese adolescents. These findings enriched our understanding of the relationship among academic stress, psychological distress, and mobile phone addiction as well as the boundary conditions of this relation from the valuable perspectives of the integration of environmental factors and psychological traits. Specifically, this study revealed that psychological distress could act as a bridge in the link between academic stress and mobile phone addiction. Besides, peer alienation and rumination, respectively, as well as their combination could moderate the relation between academic stress and psychological distress and the indirect effect of psychological distress in the link between academic stress and mobile phone addiction. These findings were helpful for us in understanding the underlying mechanisms and boundary conditions of academic stress resulting in mobile phone addiction from the perspective of multi-factor interactions.

In addition, our research offers helpful practical suggestions, particularly in education and counseling. Firstly, educators should acknowledge the impact of academic stress and negative emotions (such as anxiety and depression) on early mobile phone use [77]. By doing so, they can develop targeted interventions to prevent adolescents from becoming addicted to mobile phones. Encouraging reasonable academic expectations, monitoring mental health, and guiding students toward extracurricular activities are all effective approaches. Secondly, counselors can assist adolescents in developing positive stress coping styles, such as stress mentality [78]. By improving their ability to recognize and manage academic stress and psychological distress, enhancing social skills, and providing cognitive behavior therapy, counselors can significantly reduce mobile phone addiction symptoms. Additionally, targeted psychological intervention training and positive thinking training can help in reducing the level of contemplation in adolescents [79]. This results in slower psychological distress and prevents addiction to mobile phones during periods of academic stress.

## Supporting information

S1 Checklist STROBE statement—Checklist of items that should be included in reports of observational studies. (DOCX)

S1 Data (SAV)
