# Supplementary material for: How and when is academic stress associated with mobile phone addiction? The roles of psychological distress, peer alienation and rumination
Source: PLoS One. 2024 Feb 12;19(2):e0293094. doi: 10.1371/journal.pone.0293094 (PMC10861088; doi:10.1371/journal.pone.0293094)
Supplement: S1 Checklist — (DOCX) [file pone.0293094.s001.docx]

STROBE Statement—checklist of items that should be included in reports of observational studies

|  | Item No. | Recommendation | Page  No. | Relevant text from manuscript |
| --- | --- | --- | --- | --- |
| **Title and abstract** | 1 | (*a*) Indicate the study’s design with a commonly used term in the title or the abstract | 1 | How and when is academic stress associated with mobile phone addiction? The roles of psychological distress, peer alienation and rumination |
|  |  | (*b*) Provide in the abstract an informative and balanced summary of what was done and what was found | 2 | The study tested the mediating role of psychological distress and the moderating roles of peer alienation and rumination in the relationship between academic stress and mobile phone addiction. |
| Introduction | | | |  |
| Background/rationale | 2 | Explain the scientific background and rationale for the investigation being reported | 2-3 | Mobile phone addiction has a high detection rate among adolescents and is thought to be related to academic stress. |
| Objectives | 3 | State specific objectives, including any prespecified hypotheses | 4-9 | **Hypothesis 1**: Academic stress will positively predict mobile phone addiction.  **Hypothesis 2:** Psychological distress will mediate the association between academic stress and mobile phone addiction.  **Hypothesis 3:** The relationship among academic stress, psychological distress and mobile phone addiction may vary with individuals’ levels of peer alienation and rumination.  **Hypothesis 3a**: Peer alienation may moderate the association between academic stress and psychological distress or mobile phone addiction.  **Hypothesis 3b:** The association between academic stress and psychological distress or mobile phone addiction could be moderated by rumination.  **Hypothesis 3c:** The adverse effect of academic stress on psychological distress or mobile phone addiction may be stronger for individuals with higher levels of peer alienation and rumination than those with lower levels of peer alienation and rumination. |
| Methods | | | |  |
| Study design | 4 | Present key elements of study design early in the paper | 10 | The present study. Figure 1. The proposed moderated mediation model. |
| Setting | 5 | Describe the setting, locations, and relevant dates, including periods of recruitment, exposure, follow-up, and data collection | 10 | Both the two junior middle schools are typical junior middle schools from two cities located in central China.  Data collection was conducted from May 24 to 28, 2021. |
| Participants | 6 | (*a*) *Cohort study*—Give the eligibility criteria, and the sources and methods of selection of participants. Describe methods of follow-up  *Case-control study*—Give the eligibility criteria, and the sources and methods of case ascertainment and control selection. Give the rationale for the choice of cases and controls  *Cross-sectional study*—Give the eligibility criteria, and the sources and methods of selection of participants | 10 | The inclusion criteria of this study were full-time middle school students, who fill in within 10-30 minutes, and voluntarily participate in the survey. The exclusion criteria were that the filling time is less than 10 minutes, the answers was incomplete, and the answers was given regularly. A total of 760 middle school students from two junior middle schools were recruited to participate in the survey through convenience sampling, and 742 valid questionnaires were collected, with an effective rate of 97.63%. |
|  |  | (*b*) *Cohort study*—For matched studies, give matching criteria and number of exposed and unexposed  *Case-control study*—For matched studies, give matching criteria and the number of controls per case | no | no |
| Variables | 7 | Clearly define all outcomes, exposures, predictors, potential confounders, and effect modifiers. Give diagnostic criteria, if applicable | 11-13 | For the scoring method of variables, refer to the measurement section of the manuscript. |
| Data sources/ measurement | 8* | For each variable of interest, give sources of data and details of methods of assessment (measurement). Describe comparability of assessment methods if there is more than one group | 11-14 | For the scoring method of variables, refer to the measurement section of the manuscript. |
| Bias | 9 | Describe any efforts to address potential sources of bias | 11 | After obtaining informed consent, investigators encouraged the participants to respond truthfully, and emphasized the principles of anonymity, independence, and confidentiality of this survey. |
| Study size | 10 | Explain how the study size was arrived at | 10 | In this study, we used G*Power3.1.9.7 to calculate the sample size [58], the calculated parameters including Tails = two, Effect size = 0.2, α err prob = 0.01, Power (1-β) = 0.99, calculated sample size is 581. Considering the invalid response rate of the subjects, assuming that the invalid response rate is 20%, 581/(1-0.2) = 726 questionnaires should be sent out. |

Continued on next page

| Quantitative variables | 11 | Explain how quantitative variables were handled in the analyses. If applicable, describe which groupings were chosen and why | 11-13 | Academic stress, Psychological distress, Peer alienation, Rumination, Mobile phone Addiction |
| --- | --- | --- | --- | --- |
| Statistical methods | 12 | (*a*) Describe all statistical methods, including those used to control for confounding | 13-14 | Descriptive statistics, Pearson correlation analysis, Independent-sample t-test, One-way ANOVA, SPSS macro PROCESS (model 10), simple slopes analysis. |
|  |  | (*b*) Describe any methods used to examine subgroups and interactions | 14 | Simple slopes analysis. |
|  |  | (*c*) Explain how missing data were addressed | 10 | Eliminate missing date. |
|  |  | (*d*) *Cohort study*—If applicable, explain how loss to follow-up was addressed  *Case-control study*—If applicable, explain how matching of cases and controls was addressed  *Cross-sectional study*—If applicable, describe analytical methods taking account of sampling strategy | 10 | Convenience sampling |
|  |  | (*e*) Describe any sensitivity analyses | no | no |
| Results | | | | |
| Participants | 13* | (a) Report numbers of individuals at each stage of study—eg numbers potentially eligible, examined for eligibility, confirmed eligible, included in the study, completing follow-up, and analysed | 10-11 | The inclusion criteria of this study were full-time middle school students, who fill in within 10-30 minutes, and voluntarily participate in the survey. The exclusion criteria were that the filling time is less than 10 minutes, the answers was incomplete, and the answers was given regularly. A total of 760 middle school students from two junior middle schools were recruited to participate in the survey through convenience sampling, and 742 valid questionnaires were collected, with an effective rate of 97.63%. |
|  |  | (b) Give reasons for non-participation at each stage | no | no |
|  |  | (c) Consider use of a flow diagram | no | no |
| Descriptive data | 14* | (a) Give characteristics of study participants (eg demographic, clinical, social) and information on exposures and potential confounders | 14 | Table 1 |
|  |  | (b) Indicate number of participants with missing data for each variable of interest | no | no |
|  |  | (c) *Cohort study*—Summarise follow-up time (eg, average and total amount) | no | no |
| Outcome data | 15* | *Cohort study*—Report numbers of outcome events or summary measures over time | no | no |
|  |  | *Case-control study—*Report numbers in each exposure category, or summary measures of exposure | no | no |
|  |  | *Cross-sectional study—*Report numbers of outcome events or summary measures | 14-19 | Table1-3 |
| Main results | 16 | (*a*) Give unadjusted estimates and, if applicable, confounder-adjusted estimates and their precision (eg, 95% confidence interval). Make clear which confounders were adjusted for and why they were included | 15-19 | Because of prior evidence suggesting gender, age, grade, and years of mobile phone use were related to mobile phone addiction, we included gender, age, grade, and years of mobile phone use as control variables in testing for all of mediating and moderating models. |
|  |  | (*b*) Report category boundaries when continuous variables were categorized | no | no |
|  |  | (*c*) If relevant, consider translating estimates of relative risk into absolute risk for a meaningful time period | no | no |

Continued on next page

| Other analyses | 17 | Report other analyses done—eg analyses of subgroups and interactions, and sensitivity analyses | 15-18 | Table2, Table3 |
| --- | --- | --- | --- | --- |
| Discussion | | | | |
| Key results | 18 | Summarise key results with reference to study objectives | 19 | This study shed light on the mediating role of psychological distress and the moderating roles of peer alienation and rumination in the relation between academic stress and mobile phones addiction. The results will be helpful to reveal how academic stress results in mobile phone addiction, and when or for whom the link is stronger. This study enriched our understanding of the underlying mechanisms and its boundary conditions of academic stress linking to mobile phone addiction. This study also enlightens us that environmental factors and psychological traits should be considered simultaneously when exploring the relationship between academic stress and internalizing and externalizing problems, which also provides empirical evidence for prevention of mobile phone addiction in adolescents and interventions against this problem. |
| Limitations | 19 | Discuss limitations of the study, taking into account sources of potential bias or imprecision. Discuss both direction and magnitude of any potential bias | 24-25 | First, given that a rigorous causal relationship cannot be drawn from a cross-sectional design, the results of this study cannot be inferred in the framework of causality. Second, due to the limitation of convenient sampling of statistical samples recruited only in two junior middle schools in central China and the limitation of research funds, we were not allowed to interpret the conclusions of this study in populations with different demographic characteristics or different cultural environments. Moreover, since the convenient self-report questionnaire was employed to collect all the data of this study, the potential social desirability bias will also restrict our inferences about the results of this study. |
| Interpretation | 20 | Give a cautious overall interpretation of results considering objectives, limitations, multiplicity of analyses, results from similar studies, and other relevant evidence | 20-24 | For a detailed explanation of the results of this study, please refer to the discussion section of the manuscript. |
| Generalisability | 21 | Discuss the generalisability (external validity) of the study results | 24-25 | For the external validity of the study, please refer to the conclusion section of the manuscript. |
| Other information | |  | | |
| Funding | 22 | Give the source of funding and the role of the funders for the present study and, if applicable, for the original study on which the present article is based | 26 | This work was supported by the National Social Science Foundation of China (Grant Number: 22BSH098) and China Scholarship Council. |

*Give information separately for cases and controls in case-control studies and, if applicable, for exposed and unexposed groups in cohort and cross-sectional studies.

**Note:** An Explanation and Elaboration article discusses each checklist item and gives methodological background and published examples of transparent reporting. The STROBE checklist is best used in conjunction with this article (freely available on the Web sites of PLoS Medicine at http://www.plosmedicine.org/, Annals of Internal Medicine at http://www.annals.org/, and Epidemiology at http://www.epidem.com/). Information on the STROBE Initiative is available at www.strobe-statement.org.
